# Eight Weeks of* Cosmos caudatus* (Ulam Raja) Supplementation Improves Glycemic Status in Patients with Type 2 Diabetes: A Randomized Controlled Trial

**DOI:** 10.1155/2015/405615

**Published:** 2015-12-06

**Authors:** Shi-Hui Cheng, Amin Ismail, Joseph Anthony, Ooi Chuan Ng, Azizah Abdul Hamid, Mohd Yusof Barakatun-Nisak

**Affiliations:** ^1^Department of Nutrition and Dietetics, Faculty of Medicine and Health Sciences, Universiti Putra Malaysia, 43300 Serdang, Selangor, Malaysia; ^2^Research Centre of Excellence for Nutrition and Non-Communicable Diseases, Faculty of Medicine and Health Sciences, Universiti Putra Malaysia, 43300 Serdang, Selangor, Malaysia; ^3^Department of Medicine, Faculty of Medicine and Health Sciences, Universiti Putra Malaysia, 43300 Serdang, Selangor, Malaysia; ^4^Department of Food Science, Faculty of Food Science and Technology, Universiti Putra Malaysia, 43300 Serdang, Selangor, Malaysia

## Abstract

*Objectives*. Optimizing glycemic control is crucial to prevent type 2 diabetes related complications.* Cosmos caudatus* is reported to have promising effect in improving plasma blood glucose in an animal model. However, its impact on human remains ambiguous. This study was carried out to evaluate the effectiveness of* C. caudatus* on glycemic status in patients with type 2 diabetes.* Materials and Methods*. In this randomized controlled trial with two-arm parallel-group design, a total of 101 subjects with type 2 diabetes were randomly allocated to diabetic-ulam or diabetic controls for eight weeks. Subjects in diabetic-ulam group consumed 15 g of* C. caudatus* daily for eight weeks while diabetic controls abstained from taking* C. caudatus.* Both groups received the standard lifestyle advice.* Results*. After 8 weeks of supplementation,* C. caudatus* significantly reduced serum insulin (−1.16 versus +3.91), reduced HOMA-IR (−1.09 versus +1.34), and increased QUICKI (+0.05 versus −0.03) in diabetic-ulam group compared with the diabetic controls. HbA1C level was improved although it is not statistically significant (−0.76% versus −0.37%).* C. caudatus* was safe to consume.* Conclusions*.* C. caudatus* supplementation significantly improves insulin resistance and insulin sensitivity in patients with type 2 diabetes.

## 1. Introduction

Type 2 diabetes is a prevalent noncommunicable disease that threatens all nations. In 2014, more than 380 million people have diabetes worldwide [1]. It is well known that type 2 diabetes mellitus is a chronic disease caused by pancreatic beta cells dysfunction and insulin resistance in peripheral muscle tissues [2–4]. Owing to the pivotal role of hyperglycemia and insulin resistance in the pathogenesis of type 2 diabetes, targeting of both improving glycemic control and lowering insulin resistance would be of paramount importance. Long-term treatment with oral antidiabetic drugs is effective; however they may cause side effects such as the risk of lactic acidosis with metformin and the risk of hypoglycemia with sulphonylureas [5]. Furthermore, researchers have shown that long-term treatment with oral antidiabetic drugs is ineffective in protecting the declining function of the pancreatic beta cells [6, 7].

Medicinal plants have always been used as a traditional medicine to treat several diseases [8] and their uses are safer than the oral antidiabetic drugs [9].* Cosmos caudatus* or known locally as* ulam raja* is a medicinal herb found in tropical countries.* C. caudatus* contained a variety of bioactive compounds, such as ascorbic acid, quercetin, proanthocyanidin, chlorogenic acid, and catechin [10–12]. It has been reported previously that* C. caudatus* possess high antioxidant capacity [12–14]. Furthermore,* C. caudatus* has been shown to exhibit various medicinal properties, such as antidiabetic [15], antihypertensive [16], and anti-inflammatory [17] in animal studies. However, its clinical effect in human remains obscure. Therefore we carried out this randomized controlled trial to determine the effectiveness of 8-week* C. caudatus* supplementation on glycemic status and insulin sensitivity in patients with type 2 diabetes.

## 2. Material and Methods

### 2.1. Study Design

This single-centre, randomized, two-arm parallel controlled clinical trial was conducted at General Medical Clinic and Endocrine Clinic of Hospital Serdang, a tertiary care government hospital, Malaysia. This trial was approved by the Ethics Committee for Research Involving Human Subjects of Universiti Putra Malaysia (JKEUPM) (FPSK_Ogos (13)05), Herbal Medicine Research Centre, Institute for Medical Research Malaysia (version 1, 8/2014), and Medical Research and Ethics Committee Ministry of Health Malaysia (NMRR-13-1344-18177). This trial was also registered with clinicalTrials.gov, identifier number NCT02322268. All procedures in this trial were conducted in accordance with Helsinki Declaration and Good Clinical Practice guidelines.

### 2.2. Participants

Patients were eligible for enrolment if they were aged between 30 and 65 years, have confirmed diagnosis of type 2 diabetes for more than 6 months, have last HbA1C value greater than 7%, were treated with stabilized dose of antidiabetic drugs, and were expected to keep the dose throughout the trial. Exclusion criteria included pregnancy, insulin treatment, acute infection, severe liver disease, kidney disease and gastrointestinal disease, and anticoagulant therapy such as warfarin and aspirin. All subjects gave written informed consent before enrolment.

### 2.3. Randomization and Intervention

Subjects were randomly allocated to either diabetic-ulam group or diabetic control group using permuted block randomization in block of 4 and 6. Subjects in the diabetic-ulam group consumed 15 g of fresh* C. caudatus* daily for 8 weeks whereas diabetic controls were asked to abstain from consuming* C. caudatus*. Both groups were given the standard lifestyle interventions which include education to follow medical nutrition therapy and physical activity recommendation.

### 2.4. Study Visits

Patients were asked to visit clinical centre every 4 weeks after screening, which includes baseline, week 4 (middle of study), and week 8 (end of study). We recorded subjects' sociodemographic data at the baseline. At each follow-up, we measured subjects' weight, height, and waist circumference. Venous blood samples were drawn after an overnight fasting to measure biochemical indices. All biochemical parameters were measured in all 3 visits except HbA1C which was not measured in week 4. Patients were contacted weekly during the study period and all the occurrences of the adverse events such as loose stools, abdominal discomfort, bloating, flatulence, sign of hypoglycemia, and sign of hyperglycemia were recorded.

### 2.5. Biochemical Analysis

Fasting blood glucose was measured by the hexokinase method (Architect Ci 8200 analyzer, Abbott Laboratories, USA). HbA1C was assayed by turbidimetric inhibition immunoassay (Cobas Integra 800, Roche Diagnostics, Germany). Serum insulin level was assayed by chemiluminescent microparticle immunoassay (Architect Ci 8200 analyzer, Abbott Laboratories, USA). Homeostatic model assessment-insulin resistance (HOMA-IR) was calculated as fasting insulin (*μ*U/mL) × fasting glucose (mmol/L)/22.5 [18]. Quantitative insulin sensitivity check index (QUICKI) was calculated as 1/[log (fasting insulin in *μ*U/mL) + log (fasting glucose in mmol/L)] [19]. Lipid profile was determined using enzymatic-colorimetric method (Architect Ci 8200 analyzer, Abbott Laboratories, USA). hs-CRP was quantified using quantitative immunoturbidimetric determination (Architect Ci 8200 analyzer, Abbott Laboratories, USA). All measurements were performed by BP Clinical Laboratory.

### 2.6. Sample Size

We need to enroll 38 patients in each group to detect 1% changes in HbA1C [20] with standard deviation of 1.75% [20] at 95% confidence level and 80% power. To allow an additional dropout rate of 20%, therefore 46 subjects are needed for each group.

### 2.7. Statistical Analysis

Statistical analysis was performed using SPSS version 21 for windows (SPSS Inc., Chicago, USA). Data were expressed as mean ± SD for continuous parameters and percentage for categorical parameters. Baseline characteristics between the two groups were compared using independent *t*-test for continuous variables and chi-square test for categorical variables. All outcome measurements were evaluated based on intention to treat using the last observation carried forward imputation. Independent *t*-test was used to assess the statistical significant differences between means of the two groups at different time points. A *p* value of <0.05 was considered as statistically significant.

## 3. Results 

### 3.1. Description of the Subjects

**Figure 1:**
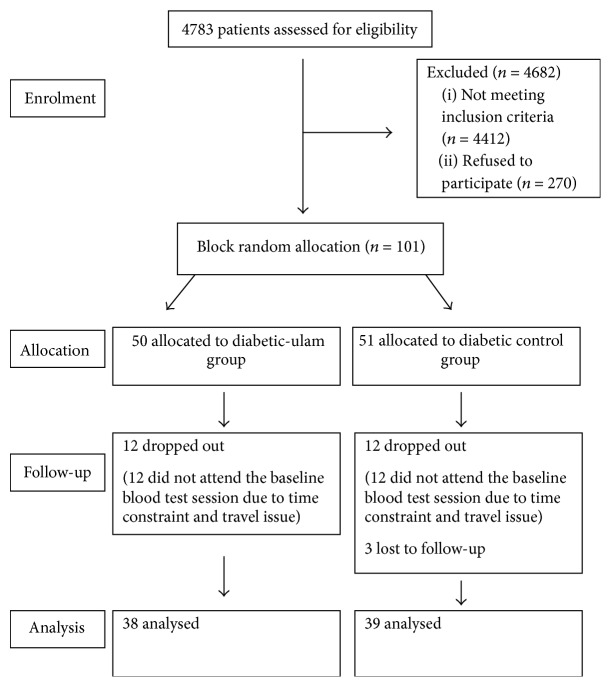
Subjects enrolment and follow-up based on CONSORT statement.

A total of 4783 subjects were initially screened in this study. After excluding noneligible subjects and those who refused to participate, a total of 101 patients with type 2 diabetes were recruited and randomly allocated to diabetic-ulam group (50 subjects) and diabetic control group (51 subjects). Patients who did not attend the baseline blood test were excluded. Three subjects from diabetic control group failed to follow up. The final analysis was performed on 77 subjects (38 diabetic-ulam group; 39 diabetic controls) using intention-to-treat analysis. A flowchart of the study trial is presented in Figure 1.

### 3.2. Baseline Characteristic

The baseline characteristics of the subjects in both groups are presented in Table 1. All parameters include sociodemographic, anthropometry, and biochemical data between diabetic-ulam group and diabetic controls were not significant at baseline. A majority of the subjects took metformin as oral antidiabetic drug and others took metformin and another oral antidiabetic drug concomitantly. However, there was no significant difference in drug treatment between the two groups (Table 1).

### 3.3. Effectiveness of* C. caudatus* on Glycemic Status, hs-CRP, and Lipid Profile

**Figure 2:**
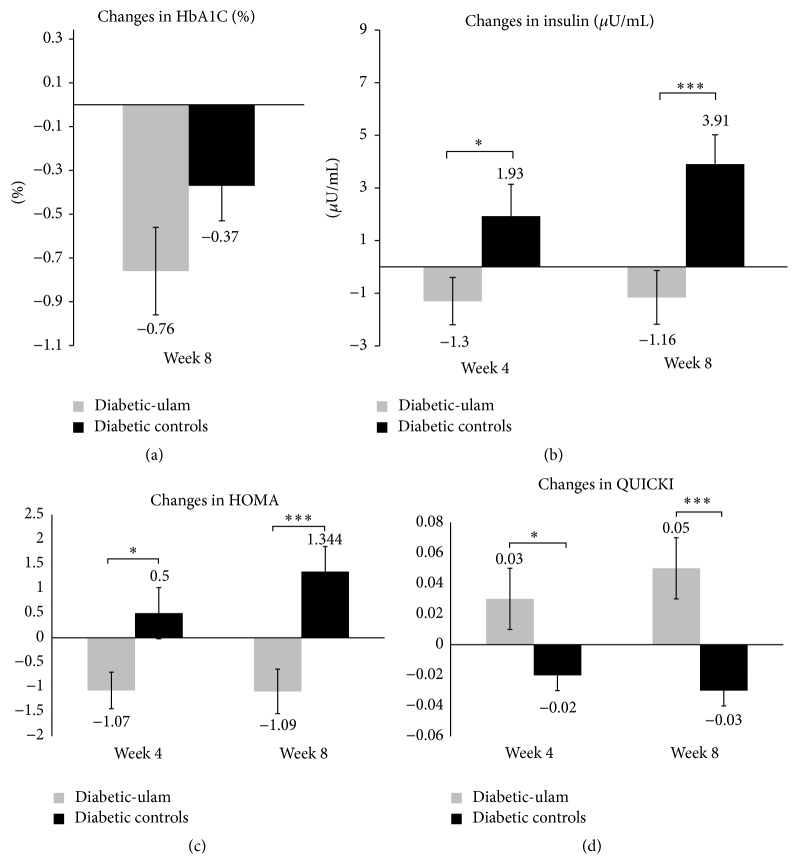
Mean difference from baseline, week 4, and week 8. (a) HbA1C. (b) Insulin IR,  ^*∗*^ *p* < 0.05,  ^*∗∗∗*^ *p* < 0.001. (c) HOMA-IR,  ^*∗*^ *p* < 0.05,  ^*∗∗∗*^ *p* < 0.001. (d) QUICKI IR,  ^*∗*^ *p* < 0.05 , ^*∗∗∗*^ *p* < 0.001.

The means of biochemical parameters on glycemic status comparing the two groups are presented in Table 2. Mean differences from baseline are illustrated in Figures 2(a)–2(d). Mean HbA1C levels were lowered in the diabetic-ulam group when compared with diabetic controls at the final visit but did not reach statistical significance (−0.76% versus −0.37%) (Figure 2(a), Table 2). Mean serum insulin levels of the diabetic-ulam group were significantly lower than those of diabetic controls at week 4 and week 8 (−1.16 versus +3.91) (Figure 2(b), Table 2).

A HOMA-IR level represents insulin resistance [18]. HOMA-IR level was found to be significantly lowered in diabetic-ulam group when compared with those in diabetic controls (−1.09 versus +1.34) (Figure 2(c), Table 2). QUICKI was measured as outcome related to insulin sensitivity [19].  Figure 2(d) and Table 2 show that QUICKI in the diabetic-ulam group was statistically elevated at week 4 and week 8 (+0.05 versus −0.03).

Levels of hs-CRP, a measurement of inflammatory marker, in the diabetic-ulam group were reduced at all follow-up visits compared to diabetic controls (Table 2). However, the reduction of the hs-CRP levels between two groups was not statistically significant. Similarly, there were no significant differences in the total cholesterol, triglycerides, HDL cholesterol, and LDL cholesterol when comparing diabetic-ulam group and diabetic controls (Table 2).

### 3.4. Adverse Effects

To determine possible adverse effect of* C. caudatus* consumption, we determine renal profile and liver function test. Results revealed that there were no significant differences in the means of AST, ALT, urea, and creatinine between diabetic-ulam and diabetic control group (Table 2). In addition, none of the subjects reported to have adverse effect throughout the study.

## 4. Discussion 

*C. caudatus *is a medicinal plant with reported potent antioxidant. Reported evidence showed that* C. caudatus* contained ascorbic acid (108.8 mg/100 mg), quercetin (51.28 mg/100 mg), chlorogenic acid (4.54 mg/100 mg), caffeic acid (3.64 mg/100 mg), and anthocyanin (0.78 mg/100 mg) [12, 21]. To the best of our knowledge, this trial is the first randomized controlled trial to determine the effectiveness of* C. caudatus* on glycemic status in patients with type 2 diabetes.

HbA1C is an important parameter to evaluate glycemic control over two to three months [22]. A high HbA1C indicates poor control of type 2 diabetes, with an increased risk of macrovascular and microvascular complications, such as cardiovascular disease, neuropathy, and nephropathy [23]. At the present study, there was no significant difference in HbA1C between diabetic-ulam group and diabetic controls. It should be noted that, however, a greater reduction of HbA1C was observed in diabetic-ulam group (−0.76%) as compared to diabetic controls (−0.37%).

Insulin resistance and insulin deficiency are two well-known key factors in the pathogenesis of type 2 diabetes [24]. In this study, we found that* C. caudatus* supplementation reduced serum fasting insulin level, improved insulin resistance indicated by a decreased HOMA-IR, and improved insulin sensitivity by an increased QUICKI. This indicated that* C. caudatus *might have a beneficial effect on insulin sensitivity and insulin resistance despite the short-term intervention period.

In addition to its beneficial effect on glycemic control,* C. caudatus* supplementation tended to reduce the hs-CRP level in diabetic-ulam group, although not statistically significant. hs-CRP, an inflammatory marker, is strongly associated with the risk of cardiovascular disease [25]. Therefore, decreased trend of hs-CRP observed in diabetic-ulam group might indicate that* C. caudatus* have a beneficial effect against cardiovascular disease. Previously, the anti-inflammatory effect of* C. caudatus* has been demonstrated by reducing prostaglandin synthesis [17].

Furthermore, it has been shown that* C. caudatus* could improve the lipid profile [15]. However, we did not observe any significant difference in the total cholesterol, LDL-c, and triglyceride levels after* C. caudatus* consumption. The discrepancies may be explained that not all the patients had dyslipidemia (61%). Another possible factor that influences the result was the use of lipid-lowering drug treatments. All the patients with dyslipidemia consumed lipid-lowering drugs.

Additionally, dietary intakes and physical activity level of the patients did not differ between two groups. There was a decrease in the energy and macronutrients intake in both groups throughout the study. However, the reduction was comparable between two groups (data not shown). Similarly, physical activity level increased in both groups. Nevertheless, the increment was comparable between groups and there were no statistically significant changes over the duration of study (data not shown). Hence, it is unlikely that dietary intake and physical activity confounded this study.

Possible mechanisms are being studied in order to explain the role of* C. caudatus* in glucose metabolism. The possible mechanism by which* C. caudatus* exerts its effect is through decreasing oxidative stress [12]. Oxidative stress affects insulin secretion and action, which in turn leads to beta cell dysfunction and insulin resistance [26–28].* C. caudatus* has been shown to have excellent antioxidant capacity [13, 14], which is beneficial in reducing the oxidative stress [12]. Besides,* C. caudatus* is found to have a good inhibitory effect against enzyme alpha-glucosidase, thus attenuates the intestinal glucose uptake, and suppresses postprandial hyperglycemia [29].

Overall, we found that an eight-week supplementation of* C. caudatus* was safe and well tolerated. None of the patients reported adverse event such as gastrointestinal disturbance after consuming* C. caudatus*. Additionally, no sign of hypoglycemia or hyperglycemia was reported among the subjects in diabetic-ulam group. Furthermore, we have not found any significant changes in the liver function and kidney function in diabetic-ulam group when compared to the diabetic controls.

However, there are limitations in this trial. Dose-dependent response evaluation is not carried out in this study. Secondly, lack of placebo used in this trial could contribute to bias in the outcomes. Besides, other inflammatory parameters such as interleukin 6 (IL-6) and tumor necrosis factor (TNF-*α*) were not measured.

## 5. Conclusions

Our results indicate that short-term* C. caudatus *supplementation is effective in improving insulin sensitivity in patients with type 2 diabetes.* C. caudatus *has the potential to develop as functional food. Given the escalating prevalence of type 2 diabetes worldwide, further clinical trials on long-term effect of* C. caudatus* in patients with type 2 diabetes are warranted.

## Figures and Tables

**Table 1:** Baseline characteristics of the study participants.

	Diabetic-ulam group (*n* = 38)	Diabetic controls (*n* = 39)	*p* value
Age (years)	48.4 ± 9.1	50.9 ± 9.1	0.213
Gender: male (%)	19 (50)	24 (61.5)	0.308
Gender: female	19 (50)	15 (38.5)	0.308
Duration of diabetes (years)	6.2 ± 4.6	7.7 ± 6.6	0.248
T2DM treatment			0.350
Metformin	26 (68.4)	23 (59)	
Metformin + SU	11 (28.9)	16 (41)	
Metformin + acarbose	1 (2.6)	0 (0)	
History of hypertension	23 (60.5)	30 (76.9)	0.120
History of dyslipidemia	23 (60.5)	24 (61.5)	0.927
Weight (kg)	77.79 ± 13.58	82.52 ± 10.72	0.144
BMI (kg/m^2^)	29.14 ± 4.94	30.41 ± 4.37	0.233
Waist circumference (cm)	98.12 ± 12.82	102.45 ± 11.14	0.117
FBG (mmol/L)	9.66 ± 3.20	8.44 ± 2.68	0.073
HbA1C (%)	8.81 ± 1.71	8.78 ± 1.40	0.942
Serum insulin (*μ*U/mL)	10.67 ± 7.42	12.73 ± 6.77	0.208
HOMA-IR	4.46 ± 3.39	3.94 ± 3.67	0.722
QUICKI	0.53 ± 0.08	0.52 ± 0.07	0.459
hs-CRP (mg/L)	4.48 ± 3.16	4.03 ± 3.14	0.591
TC (mmol/L)	5.04 ± 1.23	4.91 ± 1.39	0.680
TG (mmol/L)	2.26 ± 1.97	1.94 ± 0.91	0.370
HDL-c (mmol/L)	1.40 ± 0.31	1.45 ± 0.30	0.437
LDL-c (mmol/L)	2.82 ± 0.86	2.62 ± 1.19	0.421

T2DM: type 2 diabetes mellitus, SU: sulphonylurea, BMI: body mass index, FBG: fasting blood glucose, HbA1C: glycated hemoglobin, HOMA-IR: homeostatic model assessment-insulin resistance, QUICKI: quantitative insulin sensitivity check index, hs-CRP: high-sensitivity C-reactive protein, TC: total cholesterol, TG: triglycerides, HDL-c: high-density lipoprotein cholesterol, and LDL-c: low density lipoprotein cholesterol.

**Table 2:** Mean changes in biochemical parameters.

Parameters		Group		*p* value
		Diabetic-ulam (*n* = 38)	Diabetic controls (*n* = 39)	
		Mean ± SD	Mean ± SD	
HbA1C (%)	Baseline	8.81 ± 1.71	8.78 ± 1.40	0.942
	Week 8	8.05 ± 1.67	8.41 ± 1.40	0.302

Serum insulin (*μ*U/mL)	Baseline	10.67 ± 7.42	12.73 ± 6.77	0.208
	Week 4	9.37 ± 6.26	14.66 ± 7.36	0.002^*∗∗*^
	Week 8	9.52 ± 5.73	16.64 ± 8.04	0.001^*∗∗∗*^

HOMA	Baseline	4.46 ± 3.39	3.94 ± 3.67	0.722
	Week 4	3.58 ± 2.90	5.44 ± 3.23	0.014^*∗*^
	Week 8	3.56 ± 1.94	6.28 ± 3.71	0.001^*∗∗∗*^

QUICKI	Baseline	0.53 ± 0.08	0.52 ± 0.07	0.459
	Week 4	0.56 ± 0.09	0.50 ± 0.05	0.001^*∗∗∗*^
	Week 8	0.58 ± 0.11	0.49 ± 0.07	0.001^*∗∗∗*^

hs-CRP (mg/L)	Baseline	4.48 ± 3.16	4.03 ± 3.14	0.591
	Week 4	3.09 ± 3.04	3.65 ± 3.21	0.431
	Week 8	2.95 ± 2.68	3.79 ± 2.80	0.182

Total cholesterol (mmol/L)	Baseline	5.04 ± 1.23	4.91 ± 1.39	0.680
	Week 4	4.78 ± 1.15	4.59 ± 1.15	0.479
	Week 8	5.04 ± 1.18	4.69 ± 1.30	0.228

Triglycerides (mmol/L)	Baseline	2.26 ± 1.97	1.94 ± 0.91	0.370
	Week 4	2.01 ± 1.27	1.78 ± 0.90	0.371
	Week 8	1.97 ± 1.41	1.72 ± 0.64	0.303

HDL-c (mmol/L)	Baseline	1.40 ± 0.31	1.45 ± 0.30	0.437
	Week 4	1.37 ± 0.29	1.44 ± 0.29	0.374
	Week 8	1.46 ± 0.31	1.40 ± 0.26	0.397

LDL-c (mmol/L)	Baseline	2.82 ± 0.86	2.62 ± 1.19	0.421
	Week 4	2.54 ± 0.87	2.51 ± 1.03	0.875
	Week 8	2.49 ± 0.99	2.56 ± 1.10	0.771

AST (U/L)	Baseline	25.71 ± 13.99	25.44 ± 12.34	0.927
	Week 4	23.66 ± 9.90	26.72 ± 13.72	0.266
	Week 8	23.63 ± 10.92	25.62 ± 11.81	0.447

ALT (U/L)	Baseline	33.32 ± 21.18	31.82 ± 19.81	0.773
	Week 4	29.84 ± 18.63	34.41 ± 22.48	0.335
	Week 8	30.97 ± 19.91	33.51 ± 18.39	0.563

Urea (mmol/L)	Baseline	4.95 ± 1.49	4.71 ± 2.09	0.562
	Week 4	4.50 ± 1.42	4.52 ± 2.02	0.975
	Week 8	4.90 ± 1.61	4.38 ± 1.68	0.168

Creatinine (*μ*mol/L)	Baseline	85.42 ± 21.08	91.18 ± 22.62	0.252
	Week 4	84.53 ± 20.92	88.20 ± 23.28	0.469
	Week 8	84.53 ± 22.34	87.99 ± 21.86	0.494

^*∗*^ *p* < 0.05,  ^*∗∗*^ *p* < 0.01, and  ^*∗∗∗*^ *p* < 0.001.

SD: standard deviation, HDL-c: high-density lipoprotein cholesterol, LDL-c: low density lipoprotein cholesterol, AST: aspartate aminotransferase, and ALT: alanine aminotransferase.
